# Imaging of inflammation and regeneration: a novel trend dissecting dynamic features of biological phenomena in vivo

**DOI:** 10.1186/s41232-017-0057-2

**Published:** 2017-12-14

**Authors:** Masaru Ishii

**Affiliations:** Department of Immunology and Cell Biology, Graduate School of Medicine and Frontier Biosciences, Osaka University, Osaka, Japan

**Keywords:** Inflammation, Immunity, Regeneration, Imaging, Multi-photon microscopy, MRI

All the living creatures are continuously moving, and the regulated movement is an essential nature for animals. This also holds true for the small entities inside our body, such as cells and tissues, which are moving spontaneously or mobilized passively in the living animals. Nevertheless, conventional histological analyses could only obtain “static” information, like “snapshot”, of dynamic biological phenomenon, and we could just build imagination of their in vivo movement based on the series of snapshots. Recent advances in live imaging technologies have enabled us to grasp the real modes of cellular dynamics in vivo, and the trend has been revolutionizing the diverse fields of biomedical sciences. Among them, inflammation and regeneration, which are characterized by active migration and positioning of a diversity of hematopoietic (immune) and non-hematopoietic cell types, are well analyzed by live imaging technology revealing their dynamic natures.

In terms of optical imaging technique, invention and increased usability of multi-photon excitation microscopy was a landmark for opening a new era of this new trend in sciences. With this microscopy, we are able to see deep inside of living tissues and organs “intravitally” with less damages. Since the first reports showing the migratory behaviors of T lymphocytes and dendritic cells within lymph nodes [1, 2], a large number of studies have been reported on immune cellular dynamics in various tissues, such as skin, bone marrow, thymus, lung, liver, and nervous systems [3]. Nevertheless, most of these studies are clarifying steady-state functions of immune cells in physiological conditions, and their modes of cellular dynamics in rather pathological conditions, such as inflammation and regeneration, are currently being revealed extensively. In addition to the enormous advances in optical imaging technology, various other imaging modalities such as magnetic resonance imaging (MRI) and positron emission tomography (PET) have also been greatly developed recently, which as well dissect spatiotemporal aspects of dynamic biological phenomenon with respective unique “observation windows”, different from those of optical microscopy.

In the thematic series reviews, three topics on these issues are overviewed. First, two articles deal with inflammatory cell dynamics in peripheral tissues such as bone and skin by using intravital multiphoton microscopy techniques. Dr. Kikuta and the colleagues summarized their series of original studies showing the real modes of action of osteoclast, a bone-destroying macrophage, in inflammatory conditions, such as in arthritic joints and also the bone regenerative interaction between osteoclasts and osteoblasts, bone-forming cell types. Dr. Kabashima’s group reviewed their live imaging studies of skins revealing the control of vascular permeability for properly distributing serum proteins in normal and inflammatory conditions. The third article by Dr. Yoshioka deals with the studies by another characteristic imaging modality, MRI, for revealing immune cell dynamics in inflammatory states. Unlike the commercial MRI, which is commonly used for visualizing organ structures in clinical diagnosis, ultra-high field 11.7 T MRI enables us to see any single immune cells in total tissues and organs, irrespective of their depth from body surfaces. Even though the temporal resolution is still limited, this technology would be emerging as a new innovative tool for dissecting dynamic nature of inflammation and regeneration in future. Here, I would cordially like to express my gratitude to the distinguished researchers who kindly contributed to the special review series. I hope these reviews would provide novel insights for general readers in the field of inflammation and regeneration.

## References

[1] Stoll S, Delon J, Brotz TM, Germain RN (2002). Dynamic imaging of T cell-dendritic cell interactions in lymph nodes. Science.

[2] Miller MJ, Wei SH, Parker I, Cahalan MD (2002). Two-photon imaging of lymphocyte motility and antigen response in intact lymph node. Science.

[3] Germain RN, Robey EA, Cahalan MD (2012). A decade of imaging cellular motility and interaction dynamics in the immune system. Science.

